# The Efficacy of Omentopexy During Laparoscopic Sleeve Gastrectomy: Comparative Analysis of Surgical Outcomes, Complications, and Quality of Life

**DOI:** 10.7759/cureus.45201

**Published:** 2023-09-14

**Authors:** Suleyman Caglar Ertekin

**Affiliations:** 1 General Surgery, Private Clinic, Izmir, TUR; 2 General Surgery, Altınbas University, İstanbul, TUR

**Keywords:** gastroesophageal reflux disorder (gerd), gastrointestinal symptoms, quality-of-life, omentopexy, sleeve gastrectomy

## Abstract

Introduction: The increasing popularity of laparoscopic sleeve gastrectomy (LSG) as a bariatric procedure has been accompanied by concerns about potential complications, including staple line bleeding and leaks. Additionally, postoperative quality of life can be affected by gastroesophageal reflux disease (GERD) and food-related issues. In light of these factors, there is a need to compare the outcomes of standard LSG with LSG with omentopexy. This comparative analysis aims to provide insights into the distinct recovery processes associated with these two approaches.

Materials and methods: This retrospective study aimed to compare the outcomes of LSG in two groups: LSG alone and LSG with omentopexy. Data collected from January 2022 to April 2022 included patient characteristics, surgical details, complications, medication usage, and follow-up. Gastrointestinal Quality of Life Index (GIQLI) scores were recorded at postoperative intervals of 15 days, one month, and three months.

Results: This study analyzed 29 patients who underwent standard LSG and 36 patients who underwent LSG combined with omentopexy. The two groups exhibited similarities in terms of age, gender, body mass index (BMI), comorbidities, surgical duration, complications, hospitalization duration, and medication requirements (p > 0.005). Telephone consultations were significantly higher in the LSG with omentopexy group (p < 0.001). Nausea (p = 0.486) and vomiting (p = 0.603) rates did not significantly differ, but the constipation rate at one month was higher in LSG with omentopexy (p = 0.244). The flatulence rate at one month was significantly higher in LSG with omentopexy (p < 0.007). GIQLI scores were significantly lower in LSG with omentopexy at 15 days (p < 0.001) and one month (p < 0.001), but not at three months (p = 0.884).

Conclusion: This study demonstrates that LSG and LSG with omentopexy have similar surgical outcomes and short-term complications. However, differences exist in postoperative symptoms and quality of life experiences.

## Introduction

Laparoscopic sleeve gastrectomy (LSG) has gained significant popularity as a bariatric procedure due to its perceived procedural ease, substantial improvements in comorbidity profiles, and evident weight loss outcomes [1]. Besides facilitating satisfactory weight loss, LSG also tends to lead to improvements in various obesity-related comorbidities. Nonetheless, reports of certain potentially serious complications associated with LSG have emerged, such as staple line bleeding and leaks [2]. LSG involves resection of the gastric wall along the greater curvature. Following the procedure, patients might experience notable postoperative nausea, potentially necessitating extra clinic communication, visits, or even readmissions.

The primary complications that tend to arise after LSG encompass the aggravation of pre-existing or new-onset gastroesophageal reflux disease (GERD) and food-related issues such as constipation and flatulence [3-7]. Recent research indicates that the absence of intraabdominal ligament fixation along the greater curvature of the stomach could potentially cause misplacement of the gastric sleeve, resulting in ongoing issues with GERD and food intolerance [8,9]. These complications can exert substantial impacts on the quality of life, sometimes necessitating the transition to alternative procedures, such as a conversion to an RYGB [6]. Abdallah et al. have highlighted that implementing staple line inversion and distal fixation can effectively avert issues like staple line bleeding and axial rotation of the stomach following LSG. Similarly, the aim of applying omentopexy is to partially fix the renewed and omentum-detached stomach in its normal anatomical position, in conjunction with the colon and spleen [10].

The aim of this study is to examine and compare the postoperative complications, follow-up outcomes, and gastrointestinal complaints of patients who have undergone LSG surgery and those who have undergone combined LSG and omentopexy procedures. By doing so, we intend to gain a more comprehensive understanding of the surgical outcomes and recovery processes of patients in both groups.

## Materials and methods

This study aims to retrospectively evaluate data from patients who underwent LSG due to obesity between January 2022 and April 2022. The patients were operated on by two different surgeons with the same technique and materials. Both surgeons perform more than 100 obesity surgery procedures annually. While one surgeon performed LSG operations, the other surgeon routinely combined LSG with omentopexy. Patient information, including age, gender, preoperative body mass index (BMI), diabetes, hypertension, hyperlipidemia, fibromyalgia, anxiety disorders, length of hospital stay, surgical time (minutes), staple line leaks, staple line bleeding, postoperative GERD, as well as the postoperative pharmacological requirement for antinausea medication, including the usage of metoclopramide (mg) and ondansetron (mg), was meticulously recorded. Additionally, follow-up data encompassing clinic visit counts and readmissions related to gastrointestinal issues, constipation, and flatulence were documented. The Gastrointestinal Quality of Life Index (GIQLI) values for patients were routinely recorded at postoperative intervals of 15 days, one month, and three months [11]. These data were subsequently analyzed retrospectively.

Surgical technique

Sleeve gastrectomy procedures were conducted using a laparoscopic approach. Initially, trocars were placed in the patients' abdominal area. The gastrectomy was carried out using stomach staplers; during this phase, the stomach was meticulously dissected and the required section was removed. Upon completion of the gastrectomy, continuous sutures were applied along the stapler line using 3/0 absorbable, barbed suture material. On the other hand, in the LSG procedure with omentopexy, following the completion of the gastric resection, the entire stapler line was reinforced in conjunction with the omentum using 3/0 absorbable, barbed suture material. In the omentopexy procedure, a solitary enforcing suture was employed, and meticulous care was taken to preserve the gastroepiploic arterial and venous arcades. This strategy was aimed at minimizing the risks of hemorrhage and necrosis in the omental resection area. The subsequent steps of the procedure remained uniform for both groups.

Inclusion criteria

Patients aged 18 and older, presenting for clinical follow-up and completing GIQLI forms with a BMI ≥ 35 kg/m², were included in the study.

Exclusion criteria

Patients who did not complete their follow-up, missed their scheduled follow-up appointments, or declined to participate were excluded from the study.

Statistical analysis

A thorough statistical analysis was conducted on the baseline clinical data. Continuous data were assessed using t-tests and Mann-Whitney U analyses, whereas categorical data were analyzed through Fisher's exact test or the chi-square test. The statistical software SPSS version 22.0 (IBM Corp., Armonk, NY) was utilized for the analysis. All tests were conducted as two-tailed tests, and a significance level of P < 0.05 was employed.

## Results

The study included 29 patients who underwent LSG and 36 patients who underwent LSG with omentopexy. A mean age of 33.17 years (range: 18-65) for LSG and 34.27 years (range: 18-65) for LSG with omentopexy (p = 0.665) for the two groups showed that there were no significant age differences between them. Gender distribution demonstrated parity, with the LSG group comprising 10 males (34.5%) and 19 females (65.5%), and the LSG with omentopexy group including 13 males (36.1%) and 23 females (63.9%) (p = 0.891). BMI categorization displayed comparable patterns in both groups. In the LSG group, 34.5% fell within obese class II (BMI 35-39.9 kg/m²), while 65.5% were in obese class III (BMI ≥ 40). In the LSG with omentopexy group, 36.1% were in obese class II, and 63.9% were in obese class III (p = 0.551). Concerning comorbidities, hypertension prevalence was 13.8% in the LSG group and 25% in LSG with omentopexy (p = 0.210). Diabetes mellitus rates were 27.6% in the LSG group and 33.3% in the LSG with omentopexy (p = 0.411). Hyperlipidemia prevalence was 17.2% in LSG and 5.6% in LSG with omentopexy (p = 0.134). Moreover, fibromyalgia and anxiety disorder prevalence was slightly higher in the LSG group (24.1%) than LSG with omentopexy (13.9%), yet statistical significance was absent (p = 0.230 for both) (Table 1).

**Table 1:** Baseline characteristics of patients underwent LSG and LSG with omentopexy BMI: body mass index; LSG: laparoscopic sleeve gastrectomy

Variables	LSG (n=29)	LSG with omentopexy (n=36)	P-value
Age (years), mean ± SD, range	33.17 ± 10.09 (18–65)	34.27 ± 10.26 (18–65)	0.665
Sex, n (%)			
Male	10 (34.5)	13 (36.1)	0.891
Female	19 (65.5)	23 (63.9)	
BMI, n (%)			
Obese class II (35–39.9 kg/m^2^), n (%)	10 (34.5)	13 (36.1)	0.551
Obese class III (≥40 kg/m^2^), n (%)	19 (65.5)	23 (63.9)	
Hypertension, n (%)	4 (13.8)	9 (25)	0.210
Diabetes mellitus, n (%)	8 (27.6)	12 (33.3)	0.411
Hyperlipidemia, n (%)	5 (17.2)	2 (5.6)	0.134
Fibromyalgia, n (%)	7 (24.1)	5 (13.9)	0.230
Anxiety disorders, n (%)	7 (24.1)	5 (13.9)	0.230

Surgical duration showed no notable distinction, with LSG averaging 64.31 minutes (range: 55-86 minutes) and LSG with omentopexy averaging 64.55 minutes (range: 55-92 minutes), resulting in a non-significant difference (p = 0.900). Evaluation of postoperative complications revealed no staple line leaks in either group. Staple line bleeding occurred in 3.4% of LSG and 5.6% of LSG with omentopexy. Postoperative GERD was reported in 17.2% of the LSG group and 16.7% of the LSG with omentopexy group. Wound infections were observed in 6.9% of the LSG group and 11.1% of the LSG with omentopexy group, while wound seroma occurred in 6.9% of the LSG group and 5.6% of the LSG with omentopexy group. However, none of these differences in complications between the two groups were statistically significant (p > 0.05 for all comparisons). The length of hospital stay was comparable between the groups, with both LSG and LSG with omentopexy having a mean stay of 2.41 days (range: 2-3 days). This similarity was not statistically different (p = 0.982). Regarding the need for antinausea medication in the postoperative period, the two groups were also comparable. The LSG group had a mean need of 19.65 mg of metoclopramide (range: 10-40 mg) and 13.24 mg of ondansetron (range: 8-32 mg). The LSG with the omentopexy group had a mean need of 19.16 mg of metoclopramide (range: 10-40 mg) and 15.11 mg of ondansetron (range: 8-24 mg). These differences were not statistically significant (p = 0.796 for metoclopramide and p = 0.245 for ondansetron) (Table 2).

**Table 2:** Surgical outcomes and complications comparison between LSG and LSG with omentopexy groups GERD: gastroesophageal reflux disease; LSG: laparoscopic sleeve gastrectomy

Variables	LSG (n =29)	LSG with omentopexy (n=36)	P-value
Surgical time (min.) mean ± SD, range	64.31 ± 7.49 (55-86)	64.55 ± 7.95 (55-92)	0.900
Complications n (%)			
Staple line leaks	0	0	
Staple line bleeding	1 (3.4)	2 (5.6)	0.582
Postoperative GERD	5 (17.2)	6 (16.7)	0.603
Wound infection	2 (6.9)	4 (11.1)	0.445
Wound seroma	2 (6.9)	2 (5.6)	0.607
Length of hospital stay, days mean ± SD, range	2.41 ± 0.50 (2-3)	2.41 ± 0.50 (2-3)	0.982
Pharmacological need for antinausea medication before discharge in the postoperative period, mean ± SD, range			
Metoclopramide (mg)	19.65 ± 7.78 (10-40)	19.16 ± 7.31 (10-40)	0.796
Ondansetron (mg)	13.24 ± 6.15 (8-32)	15.11 ± 6.56 (8-24)	0.245

The average clinic visit counts within the first month were 2.20 (mean) ± 0.41 (SD) with a range of 2-3 for the LSG group (n = 29) and 2.34 (mean) ± 0.48 (SD) with a range of 2-3 for the LSG with omentopexy group (n = 36). The difference in clinic visit counts between the two groups was not statistically significant (p = 0.180). Regarding readmissions related to gastrointestinal issues, the LSG group had an average of 0.03 (mean) ± 0.18 (SD) with a range of 0-1, while the LSG with omentopexy group had an average of 0.05 (mean) ± 0.23 (SD) with a range of 0-1. No statistically significant difference was observed between the two groups in terms of readmissions related to gastrointestinal issues (p = 0.693). Telephone consultations during the first month post-surgery were analyzed, revealing an average of 2.34 (mean) ± 0.48 (SD) with a range of 2-3 for the LSG group and a notably higher average of 3.61 (mean) ± 1.45 (SD) with a range of 2-7 for the LSG with omentopexy group. This discrepancy was statistically significant (p < 0.001), suggesting that the LSG with omentopexy required more telephone consultations. The occurrence of specific symptoms was also evaluated. Nausea was reported by 17.2% of the LSG group and 13.9% of the LSG with omentopexy group, with no significant difference between the groups (p = 0.486). Similarly, vomiting was observed in 17.2% of the LSG group and 16.7% of the LSG with omentopexy group, showing no statistically significant difference (p = 0.603). However, constipation presented a contrasting scenario, with a rate of 20.7% in the LSG group and a notably higher rate of 47.2% in the LSG with omentopexy group. Despite the trend, this difference did not achieve statistical significance (p = 0.244). Lastly, flatulence exhibited a significant discrepancy between the groups. The LSG group had a flatulence rate of 13.8%, whereas the LSG with omentopexy group showed a considerably higher rate of 44.4% (p < 0.007) (Table 3).

**Table 3:** Clinical and quality of life outcomes in LSG and LSG with omentopexy groups at one month LSG: laparoscopic sleeve gastrectomy

Variables	LSG (n=29	LSG with omentopexy (n=36)	P-value
Clinic visit counts, mean ± SD, range	2.20 ± 0.41 (2–3)	2.34 ± 0.48 (2-3)	0.180
Readmissions related to gastrointestinal issues, mean ± SD, range	0.03 ± 0.18 (0-1)	0.05 ± 0.23 (0-1)	0.693
Telephone consultation, mean ± SD, range	2.34 ± 0.48 (2-3)	3.61 ± 1.45 (2-7)	<0.001
Nausea, n (%)	5 (17.2)	5 (13.9)	0.486
Vomiting, n (%)	5 (17.2)	6 (16.7)	0.603
Constipation, n (%)	6 (20.7)	17 (47.2)	0.244
Flatulence, n (%)	4 (13.8)	16 (44.4)	<0.001

The GIQLI scores were meticulously evaluated at three distinct time points post-surgery: the 15th day, one month, and three months. At the 15th day postoperative mark, the LSG group (n = 29) displayed a mean GIQLI score of 81.55 ± 7.89 (range: 65-102), while the LSG with omentopexy group (n = 36) exhibited a mean score of 73 ± 9.82 (range: 54-90). This disparity was statistically significant (p < 0.001), underscoring a notable divergence in gastrointestinal quality of life experiences shortly after the surgical interventions. Upon reaching the one-month postoperative milestone, the LSG group revealed a mean GIQLI score of 98.72 ± 8.13 (range: 85-111), whereas the LSG with omentopexy group showed a mean score of 82.41 ± 8.78 (range: 65-110). Once again, this difference in GIQLI scores was statistically significant (p < 0.001), indicating distinct outcomes in gastrointestinal quality of life experiences at this juncture. However, as time progressed to the three-month mark, the discrepancy in GIQLI scores between the two groups became negligible. The LSG group recorded a mean GIQLI score of 98.10 ± 7.33 (range: 85-111), while the LSG with omentopexy group achieved a mean score of 97.83 ± 7.45 (range: 85-111). Notably, there was no statistically significant difference in GIQLI scores between the two groups at this later time point (p = 0.884). This finding suggests a potential convergence in gastrointestinal quality of life outcomes over time, highlighting the importance of longitudinal evaluation (Table 4).

**Table 4:** GIQLI scores in LSG and LSG with omentopexy groups at 15th day, 1 month, and 3 month GIQLI: Gastrointestinal Quality of Life Index

Variables	LSG (n=29)	LSG with omentopexy (n=36)	P-value
Postoperative 15th day GIQLI scores mean ± SD, range	81.55 ± 7.89 (65–102)	73 ± 9.82 (54–90)	<0.001
Postoperative 1 month GIQLI scores mean ± SD, range	98.72 ± 8.13 (85–111)	82.41 ± 8.78 (65–110)	<0.001
Postoperative 3 month GIQLI scores mean ± SD, range	98.10 ± 7.33 (85–111)	97.83 ± 7.45 (85–111)	0.884

## Discussion

The quality of life and well-being of patients hold critical significance in evaluating surgical interventions. In this context, when selecting and implementing surgical approaches, consideration of the gastrointestinal symptoms that patients may encounter in the postoperative period is crucial. Particularly, symptoms such as nausea, vomiting, constipation, and bloating can influence patients' daily lives and diminish their quality of life. Therefore, comprehending the potential effects of surgical additions in greater detail becomes essential, as it can contribute to both the surgical decision-making process and the patients' ability to derive more favorable outcomes.

The results of our study have demonstrated that the addition of omentopexy to LSG in obese patients does not possess a significant ability to rectify food intolerance and GERD profiles after surgery. Furthermore, it reveals that patients who underwent omentopexy alongside LSG did not exhibit a notable difference in the perioperative requirement for antiemetic medication compared to those who underwent LSG without omentopexy. Nevertheless, existing studies support the hypothesis that the loss of intraabdominal ligament fixation of the greater curvature of the stomach can lead to mispositioning of the gastric sleeve, resulting in persistent GERD and food intolerance [8,9].

However, the data obtained from our study present a different conclusion rather than supporting this hypothesis. Additionally, a study in the literature aligns with the results of our study, indicating that the addition of omentopexy does not significantly improve food intolerance and GERD profiles following LSG. When focusing on clinic visit counts, no significant difference in clinic visit frequencies was observed between the LSG and LSG with omentopexy groups. This suggests that both surgical approaches might have a similar impact on patients' clinical follow-up needs. Analysis of readmissions related to gastrointestinal issues also did not reveal a significant difference between the LSG and LSG with omentopexy groups. This finding might imply that postoperative complications occurred at a similar frequency between the two groups, consistent with the literature [12].

Furthermore, the outcomes concerning telephone consultations indicate that the LSG with omentopexy group sought more frequent telephonic counseling. This trend might be attributed to a substantial increase in the incidence of constipation and bloating symptoms in the group that underwent omentopexy. This finding suggests that the omentopexy procedure could potentially exacerbate the frequency of these two gastrointestinal symptoms, leading to an augmented demand for telephone consultations. It can be postulated that in patients undergoing LSG with omentopexy, the procedure may amplify complaints of gas and constipation due to the extra traction on the transverse colon caused by omentopexy. As existing studies predominantly concentrate on postoperative GERD complaints, we have not encountered research that either corroborates or refutes our findings concerning constipation and gas complaints [13-15].

When focusing on assessments of quality of life, our study observed that postoperative 15-day and 1-month GIQLI scores were higher in the LSG group. However, when examining the three-month scores, no significant difference was noted between the groups. Existing literature on quality-of-life assessments following LSG indicates that studies have shown an improvement in patients' quality of life outcomes in the long term along with weight loss [16,17]. A potential etiology for the observed outcomes may involve early-stage partial or central ischemia of the omentum, consequent to manipulation of the gastroepiploic vascular arc during omentopexy. This ischemic condition is presumed to resolve naturally over time through revascularization mechanisms. Significantly, our study indicates that even if there is a transient impact on omental vascularity, it neither affects long-term clinical outcomes nor likely contributes to subsequent complications.

In particular, we did not come across a study that compared LSG and LSG with omentopexy procedures using scoring systems for gastrointestinal complaints and quality of life other than GERD. The early-term results of our study suggest that lower quality of life scores in the LSG with omentopexy group might be due to a higher prevalence of symptoms such as constipation and bloating. This situation might imply that omentopexy could lead to an increase in certain postoperative gastrointestinal discomforts in some patients. On the other hand, the lack of a significant difference in the quality of life scores at three months between the groups might suggest that weight loss and the establishment of patients' dietary habits could contribute to an improvement in quality of life as complaints diminish proportionally.

Nevertheless, the outcomes of this study are limited by its single-center design and lack of long-term follow-up. Further investigations with larger sample sizes and extended follow-up durations are warranted to validate these findings.

## Conclusions

Our study suggests that the addition of omentopexy to laparoscopic sleeve gastrectomy does not significantly improve gastrointestinal symptoms or food intolerance profiles in obese patients. While the short-term quality of life may be adversely impacted by symptoms such as constipation and bloating, long-term outcomes appear comparable between both surgical approaches. These findings emphasize the need for further, more extensive research to fully understand the potential advantages and disadvantages of incorporating omentopexy into laparoscopic sleeve gastrectomy.
